# Continuous Near-Infrared Spectroscopy as a Real-Time Monitor of Intravenous Thrombolysis in Acute Ischaemic Stroke: A Prospective Observational Emergency Department Study

**DOI:** 10.3390/diagnostics16213469

**Published:** 2026-10-23

**Authors:** Emre Şancı, Feride Fulya Ercan, Hamza Çıldır, Başak Bayram, Adile Öniz Özgören, Kürşad Kutluk, Neşe Çolak

**Affiliations:** 1 Department of Emergency Medicine, İzmir Bakircay University Cigli Training and Research Hospital, 35620 Izmir, Türkiye; 2 Department of Emergency Medicine, Mediclinic Middle East City Hospital, Dubai 505004, United Arab Emirates; 3 Department of Emergency Medicine, Buca Seyfi Demirsoy Training and Research Hospital, 35390 Izmir, Türkiye; 4 İzmir Eşrefpaşa Metropolitan Municipality Hospital, 35170 Izmir, Türkiye; 5 Brain and Conscious States Research Center, Near East University, Cyprus, 99138 Mersin, Türkiye; 6 Independent Researcher, 35340 Izmir, Türkiye; 7 Department of Emergency Medicine, University of Health Sciences, Tepecik Training and Research Hospital, 35020 Izmir, Türkiye

**Keywords:** acute ischaemic stroke, near-infrared spectroscopy, cerebral oximetry, intravenous thrombolysis, alteplase, neuromonitoring, emergency medicine

## Abstract

**Background.** Intravenous (IV) thrombolysis is the standard of care for acute ischaemic stroke (AIS), yet there is still no objective bedside tool for monitoring the brain continuously during the infusion. Near-infrared spectroscopy (NIRS) provides non-invasive, continuous measurement of regional cerebral oxygen saturation (rSO_2_). We evaluated whether bilateral frontal NIRS, and in particular the absolute interhemispheric difference in rSO_2_ (ΔNIRS), tracks the clinical trajectory of patients receiving IV alteplase in the emergency department (ED). **Methods.** A prospective observational cohort study was conducted in the ED of a tertiary university hospital. Consecutive adults receiving IV alteplase for AIS were screened, and those enrolled underwent continuous bilateral frontal NIRS monitoring. Bilateral rSO_2_, ΔNIRS, vital signs, Glasgow Coma Scale (GCS) and National Institutes of Health Stroke Scale (NIHSS) were recorded at baseline and at 0, 15, 30, 45, 60 and 120 min after the start of the infusion. Patients were classified by clinical trajectory during monitoring as improvement, no change, or deterioration. **Results.** Forty-one patients were analyzed (26 female, 63.4%; mean age 73.6 ± 14.8 years); 15 (36.6%) improved, 21 (51.2%) showed no change and 5 (12.2%) deteriorated. In the whole cohort, absolute rSO_2_ rose only slightly in the affected hemisphere and did not change in the unaffected hemisphere. However, affected-hemisphere rSO_2_ rose significantly in the improvement group (median 59 to 67; *p* = 0.001) and fell significantly in the deterioration group (*p* = 0.015). ΔNIRS narrowed from a median of 4 (IQR 3–9) at baseline to 1 (IQR 0–5) at 120 min in the improvement group (*p* = 0.034) and did not change significantly in the other groups. The two patients with acute complications desaturated bilaterally. NIHSS correlated strongly and inversely with GCS at every time point (ρ = −0.74 to −0.84; all *p* < 0.001). **Conclusions.** During IV thrombolysis in the ED, continuous bilateral frontal NIRS tracked the clinical trajectory. Because recanalisation and reperfusion were not assessed independently, what NIRS paralleled was the clinical course, not reperfusion itself. These hypothesis-generating findings warrant larger studies that validate ΔNIRS against perfusion or vascular imaging.

## 1. Introduction

Acute ischaemic stroke (AIS) is a time-critical neurological emergency in which a sudden reduction in regional cerebral perfusion compromises neuronal function and, if prolonged, results in irreversible brain injury. Intravenous (IV) administration of recombinant tissue plasminogen activator (rt-PA) within 4.5 h of symptom onset remains a cornerstone of acute reperfusion therapy, and its benefit is strongly time-dependent [1,2]. Once the thrombolytic infusion has begun, however, emergency physicians lack an objective, continuous bedside method for monitoring the brain during treatment. Clinical assessment relies on serial neurological examinations using the National Institutes of Health Stroke Scale (NIHSS) and the Glasgow Coma Scale (GCS) [3,4], which are intermittent and examiner-dependent, and which register recovery only after neurological improvement has become clinically evident.

Near-infrared spectroscopy (NIRS) is a non-invasive optical technique that continuously estimates regional cerebral tissue oxygen saturation (rSO_2_) from the differential absorption of near-infrared light by oxyhaemoglobin and deoxyhaemoglobin [5,6]. It requires only adhesive forehead optodes, imposes no interruption of care, and has been used for decades in cardiac and neurocritical care [7]. In stroke, it has been used to study cerebral oxygenation and haemodynamics from the hyperacute phase through to rehabilitation [8,9].

Most of the clinical NIRS literature in acute stroke has been generated in the angiography suite during endovascular thrombectomy. A small series showed that successful recanalisation was accompanied by a rise in rSO_2_ over the infarcted hemisphere and by a narrowing of the interhemispheric difference, whereas a failed procedure showed neither [10]; in a larger cohort, however, sustained increases after recanalisation were seen in only a minority of patients [11]. A recent post hoc analysis of a randomised trial confirmed the pattern in 66 patients monitored during thrombectomy, although the effects were small [12]. Other groups have been unable to detect interhemispheric change, particularly in distal or non-large-vessel occlusions [13], and such negative findings have been attributed to optode placement, extracranial contamination and collateral flow [12]. More recent prospective data showed no interhemispheric or intrahemispheric rSO_2_ differences during thrombectomy, even in proximal anterior circulation occlusions, most likely because of signal contamination from surrounding tissues [14]. Time-domain NIRS has also demonstrated that haemoglobin species and tissue oxygen saturation vary according to recanalisation status, supporting the use of optical monitoring as a surrogate marker of tissue metabolic recovery following reperfusion [15]. By contrast, continuous NIRS during IV thrombolysis in the emergency department (ED), where most patients worldwide receive reperfusion therapy, remains sparsely characterised, with only a handful of small studies published to date [16,17,18].

Absolute cerebral oximetry varies substantially between individuals because of anatomical and physiological factors, including age, sex, haemoglobin concentration, extracranial tissue thickness, skull characteristics and baseline cerebrovascular status [19,20,21]. An interhemispheric difference (ΔNIRS) may therefore provide a more robust within-patient physiological marker. Our study differs from earlier work in four respects. First, the recordings were made in the ED during IV thrombolysis, without the endovascular manipulation, sedation or general anaesthesia that accompany thrombectomy, all of which can alter cerebral oxygenation in their own right. Second, we examined the full spectrum of clinical trajectories. Third, each hemisphere was analysed on its own as well as against the other. Fourth, we describe how rSO_2_ behaved around acute intra-treatment complications.

We hypothesised that the dynamic interhemispheric difference in cerebral oxygenation (ΔNIRS, expressed as an absolute value irrespective of which hemisphere is lower), rather than absolute rSO_2_, would provide an immediate and objective correlate of thrombolytic success or failure, because it is internally controlled for the large inter-individual variability in baseline cerebral oximetry. With no angiographic or perfusion-imaging reference available during thrombolysis in the ED, we tested this hypothesis against the patients’ clinical trajectory. We therefore evaluated bilateral frontal NIRS during IV alteplase administration in the ED, examining its relationship to the clinical trajectory and describing its behaviour around acute intra-treatment complications.

## 2. Methods

### 2.1. Study Design, Setting and Ethics

We conducted a prospective, single-centre observational cohort study in the ED of Dokuz Eylül University Hospital, a tertiary academic centre in İzmir, Türkiye, between November 2017 and October 2018. The study was approved by the Institutional Ethics Committee of Dokuz Eylül University (405-SBKAEK, 2017/17-06) and was registered at ClinicalTrials.gov (NCT03621917). Written informed consent was obtained from every patient or, where the patient was unable to consent, from a legally authorised representative. The study was funded by the Department of Scientific Research Projects of Dokuz Eylül University (grant 2018.KB.SAG.062). The funder had no role in the design, conduct, analysis or reporting of the study. The manuscript is reported in accordance with the STROBE statement for observational studies. Monitoring was strictly observational: no rSO_2_ value was communicated to the treating team, and no clinical decision was made on the basis of NIRS data.

### 2.2. Participants

**Figure 1:**
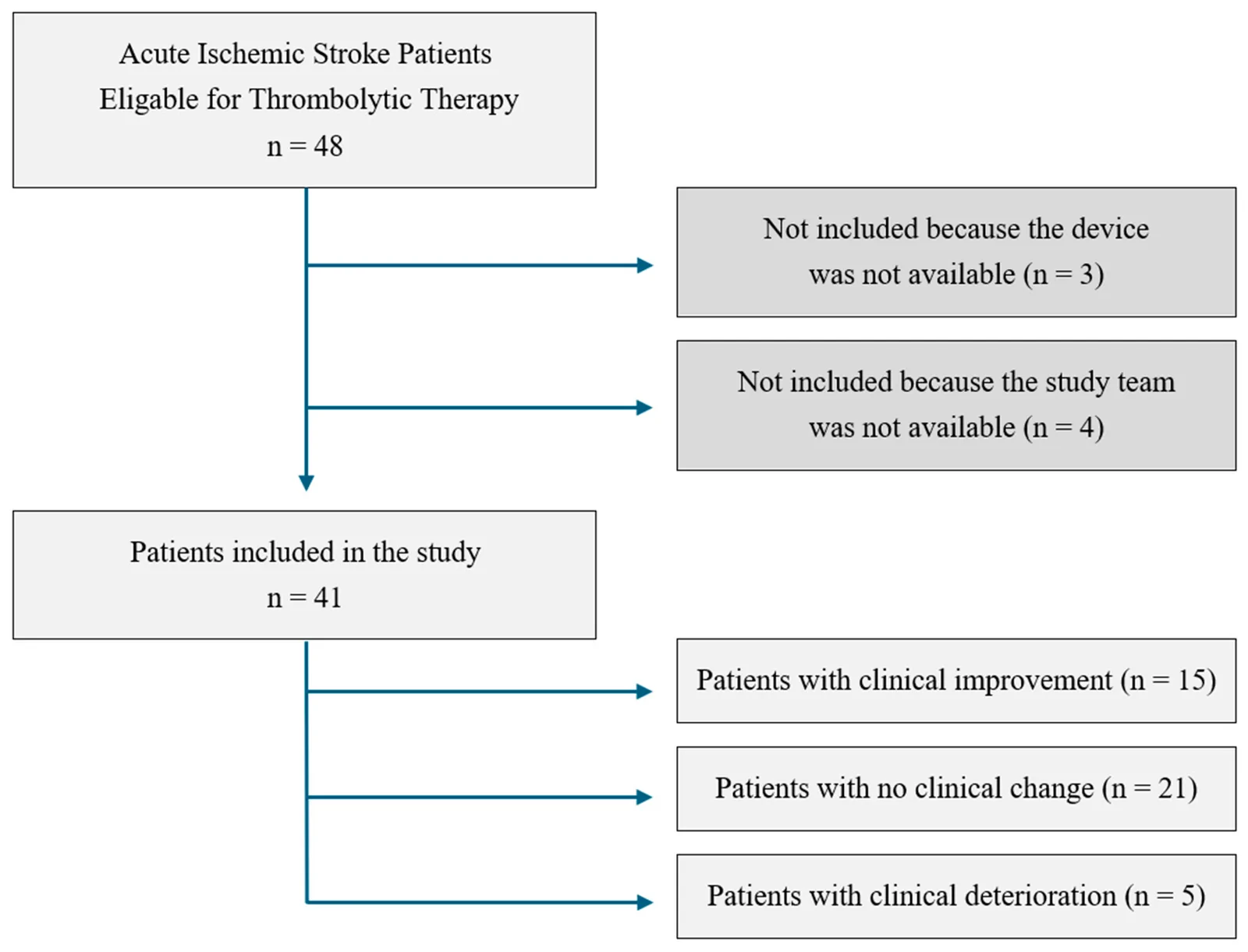
Flow diagram of patient screening and inclusion, with the clinical trajectory groups assigned during the 120-min monitoring window.

All consecutive patients aged 18 years or older who received IV alteplase for AIS in the ED during the study period were screened for eligibility. Patients were excluded if written informed consent could not be obtained, if continuous NIRS monitoring could not be performed for logistical or technical reasons (device unavailable, sensors unavailable, or no trained study investigator present), if the forehead skin at the optode site was not intact (haematoma, mass, laceration), or if clinically apparent jaundice was present since bilirubin absorbs in the near-infrared range and may distort the optical signal. Logistical exclusion depended only on whether trained staff and equipment were available at the time of presentation, not on any clinical characteristic of the patient. The study flow diagram is shown in Figure 1.

### 2.3. Neuromonitoring and Data Collection

All patients received standard continuous cardiorespiratory monitoring as part of routine thrombolysis care. Bilateral frontal cerebral oximetry was performed with an INVOS 5100C cerebral/somatic oximeter (Covidien–Medtronic, Boulder, CO, USA) using disposable adult SomaSensor optodes applied to the right and left forehead above the supraorbital ridge, lateral to the midline, and away from the sagittal sinus. The hemisphere containing the acute infarct—determined by the treating neurologist from the clinical syndrome and the admission cranial computed tomography (CT)—was designated the affected hemisphere and the contralateral side the unaffected hemisphere. The routine 24-h follow-up CT confirmed this designation in every patient. Lateralisation was never in doubt, and no patient was excluded or reassigned on these grounds. The vascular territory (anterior, middle or posterior cerebral artery) was recorded. Infarcts outside the middle cerebral artery territory, posterior cerebral artery infarcts among them, were lateralised in the same way and kept in the analysis.

Systolic and diastolic blood pressure, heart rate, respiratory rate, peripheral oxygen saturation, GCS and NIHSS were recorded together with bilateral rSO_2_ at baseline (ED arrival, before treatment) and at 0 (start of the alteplase bolus), 15, 30, 45, 60 and 120 min. ΔNIRS was calculated at each time point as the absolute difference between the rSO_2_ of the unaffected and that of the affected hemisphere (|unaffected—affected|) so that it reflects the size of the interhemispheric gap irrespective of which hemisphere has the lower value. NIRS was recorded continuously throughout so that events occurring between scheduled time points were captured. To avoid ambiguity, we used NIRS throughout for the monitoring technique, rSO_2_ for the regional cerebral oxygen saturation it measures, and ΔNIRS for the absolute interhemispheric difference in rSO_2_.

We took several steps to limit bias. Every measurement was obtained with the same device and sensor type from a standardised optode position at predefined time points. The treating team was blinded to the rSO_2_ values, which played no part in any clinical decision (Section 2.1). The criteria used to classify the clinical trajectory were prespecified in the study protocol (Section 2.4).

### 2.4. Outcome Definitions

The prespecified exposure of interest was time from the start of thrombolysis; the primary outcome measure was ΔNIRS. Patients were classified during the 120-min monitoring window into one of three mutually exclusive clinical trajectory groups, using criteria prespecified in the study protocol:

- Clinical improvement—a decrease in NIHSS of more than one point, or an unequivocal improvement in GCS or in motor function.
- Clinical deterioration—an increase in NIHSS of more than one point, or the occurrence of symptomatic haemorrhage, acute seizure, or cardiorespiratory arrest.
- No clinical change—a stable neurological examination with neither improvement nor decline.

These categories reflect the three courses that a treating physician has to recognise while the infusion is running. We preferred a three-way classification to a dichotomy so that the NIRS signal could be examined separately in patients who deteriorated, in whom we expected changes in the opposite direction to those seen in improving patients. There is no consensus definition of early neurological change. We therefore set the threshold at more than one NIHSS point (i.e., a change of at least two points), because an increase of this size has been shown to be a sensitive indicator of poor outcome and in-hospital mortality [22]. The more conservative four-point threshold generally used for deterioration within 24 h of reperfusion therapy [23] was, in our view, too insensitive for a 120-min window. For symmetry, the same threshold was applied in both directions.

### 2.5. Statistical Analysis

Analyses were performed with SPSS 25.0 (IBM Corp., Armonk, NY, USA). Normality was assessed with the Shapiro–Wilk test. Normally distributed continuous variables (age and vital signs) are presented as mean ± standard deviation (minimum–maximum) and non-normally distributed or ordinal variables (GCS, NIHSS, process-of-care intervals, rSO_2_ and ΔNIRS) as median (interquartile range, IQR). Categorical variables are presented as counts and percentages and were compared with the Pearson chi-square test or with Fisher’s exact test (Freeman–Halton extension for tables larger than 2 × 2) where expected cell counts were below five. Baseline age and vital signs were compared between the three groups with Welch’s analysis of variance, which does not assume equal variances, and age was compared between women and men with Student’s *t*-test.

Repeated measurements of vital signs across the seven time points were compared in the whole cohort and within the groups using the general linear model for repeated measures, with time as the within-subject factor; because Mauchly’s test showed that the assumption of sphericity was violated, the Greenhouse–Geisser correction was applied. The remaining variables were analysed non-parametrically: changes in rSO_2_ across the seven time points, in the whole cohort and within each group with the Friedman test; between-group comparisons of baseline GCS, NIHSS and process-of-care intervals, and of rSO_2_ at each time point with the Kruskal–Wallis test; and the change in ΔNIRS between baseline and 120 min with the Wilcoxon signed-rank test. In patients whose clinical status changed, rSO_2_ averaged over the period before and over the period after the clinical change was calculated for each patient, and the two values were compared with the Wilcoxon signed-rank test. The association between GCS and NIHSS was assessed with Spearman’s rank correlation coefficient (ρ).

Two-sided *p* values below 0.05 were considered statistically significant. Given the exploratory nature of the study and the number of comparisons across time points and groups, *p* values are reported without adjustment for multiple comparisons and should be interpreted as hypothesis-generating. No a priori sample size calculation was performed; all consecutive eligible patients treated during the study period were screened, so the length of the study period set the sample size.

## 3. Results

### 3.1. Participants and Baseline Characteristics

During the study period, 48 patients received IV thrombolysis for AIS in the ED. Seven were not enrolled (four because no trained study investigator was available and three because the NIRS device was unavailable), leaving 41 patients (85.4%) for analysis (Figure 1). There was no attrition. All 41 patients completed the 120-min monitoring protocol, with no missing rSO_2_, vital-sign or neurological-score values at any scheduled time point. Twenty-six patients (63.4%) were women and 15 (36.6%) were men; the mean age was 73.6 ± 14.8 years (range 33–95). Women were significantly older than men (77.5 ± 15.0 vs. 66.9 ± 12.3 years; *p* = 0.026).

Fifteen patients (36.6%) improved clinically during monitoring, 21 (51.2%) showed no clinical change, and 5 (12.2%) deteriorated. Of the five patients who deteriorated, three met the NIHSS criterion alone and two were classified on the basis of an acute event (one seizure, one respiratory arrest). None was classified on the basis of symptomatic haemorrhage. Of the 15 patients who improved, 13 met the NIHSS criterion and two were classified on the alternative criterion of an unequivocal improvement in GCS or motor function. Baseline characteristics and process-of-care intervals are shown in Table 1. Median door-to-imaging time was 22 min (IQR 16–31.5), and median door-to-needle time was 59 min (IQR 49.5–74); neither differed significantly between the three groups (Table 1). The middle cerebral artery territory was involved in 32 of 41 patients (78.0%); in the remaining nine, the infarct lay in the anterior (*n* = 5) or posterior (*n* = 4) cerebral artery territory.

### 3.2. Haemodynamic and Respiratory Stability

Systolic and diastolic blood pressure, heart rate, respiratory rate and peripheral oxygen saturation did not change significantly across the seven time points, either in the cohort as a whole (Table 2) or within any of the three groups. Group-specific vital signs at every time point are provided in Supplementary Table S1.

### 3.3. Neurological Scores

NIHSS and GCS were strongly and inversely correlated at every time point across the whole cohort (baseline ρ = −0.742; 0 min ρ = −0.742; 15 min ρ = −0.800; 30 min ρ = −0.809; 45 min ρ = −0.748; 60 min ρ = −0.844; 120 min ρ = −0.820; all *p* < 0.001), supporting the consistency of the neurological assessments used to define the groups. Median GCS and NIHSS by group and time point are given in Supplementary Table S2.

### 3.4. Cerebral Oxygenation by Clinical Trajectory

In the cohort analysed as a whole, absolute rSO_2_ changed little over the 120-min window: the median affected-hemisphere value rose slightly, from 59 (IQR 51–64) at baseline to 61 (IQR 52–67) at 120 min (*p* = 0.029), and the unaffected-hemisphere value was 62 (IQR 53–67.5) and 62 (IQR 55.5–66.5), respectively (*p* = 0.786); values by clinical trajectory are shown in Table 3.

Improvement group (*n* = 15). Affected-hemisphere rSO_2_ rose progressively and significantly over the treatment window, from a median of 59 (IQR 57–68) at baseline to 67 (IQR 61–70) at 120 min (*p* = 0.001), while the unaffected hemisphere remained unchanged (66 to 65; *p* = 0.939). Comparison of the mean affected-hemisphere rSO_2_ before and after the clinical change confirmed a significant post-change increase (*p* = 0.008).

Deterioration group (*n* = 5). Affected-hemisphere rSO_2_ fell significantly across the same window, from 60 (IQR 53.5–70) at baseline to 55 (IQR 51.5–64.5) at 120 min, with a nadir of 47 (IQR 32.5–61) at the 60-min time point (*p* = 0.015); the 60-min values include readings taken during one patient’s respiratory arrest (Section 3.6). The mean affected-hemisphere rSO_2_ after the clinical change was significantly lower (*p* = 0.04). With only five patients in this group, these results need to be read with caution.

No-change group (*n* = 21). Neither the affected hemisphere (56 to 57), the unaffected hemisphere (57 to 58), nor ΔNIRS changed significantly at any time point (Table 3 and Table 4).

The three groups also differed from one another in the *unaffected* hemisphere: median rSO_2_ of the structurally uninvolved hemisphere was consistently highest in patients who went on to improve and lowest, or joint lowest, in those who deteriorated, and this between-group difference was significant at every time point (*p* = 0.003 to 0.035; Table 3). Absolute cerebral oximetry values therefore carry a global, patient-level component that is unrelated to the ischaemic lesion itself, which is the principal reason we regard the internally controlled ΔNIRS as the more interpretable signal.

### 3.5. Interhemispheric Difference (ΔNIRS)

ΔNIRS (the absolute difference between the unaffected and affected hemispheres) is shown in Table 4. In the improvement group, ΔNIRS narrowed significantly, from a median of 4 (IQR 3–9) at baseline to 1 (IQR 0–5) at 120 min (*p* = 0.034), indicating equalisation of oxygenation between the two hemispheres by the end of the monitoring period. In the deterioration group, ΔNIRS did not change significantly (median 4 [IQR 1–13.5] at baseline and 5 [IQR 1–9.5] at 120 min; *p* = 0.285). ΔNIRS did not change significantly in the no-change group (*p* = 0.886) or in the cohort as a whole (*p* = 0.114).

### 3.6. Cerebral Oxygenation Around Acute Complications

Five patients deteriorated during monitoring. Two of them sustained a major acute adverse event within the monitoring window: one had a partial seizure followed by a generalised tonic–clonic seizure at the 45th minute of treatment, and the other had a respiratory arrest at the 60-min time point and was resuscitated. Both patients remained in the deterioration group and in every analysis. Their scheduled measurements were all included, even those recorded during and after the event, and for the before-and-after comparison of rSO_2_ the time of the event was taken as the time of clinical change. In both patients, a fall in rSO_2_ was recorded before the event became clinically apparent, and the desaturation was bilateral, a pattern more consistent with a systemic or global cerebral insult than with the strictly lateralised changes associated with reperfusion. The remaining three patients in this group deteriorated by NIHSS alone; in two of them, affected-hemisphere rSO_2_ fell at the time point at which the NIHSS increase was documented, and in the third it fell at the next time point. With so few patients, these observations are descriptive only.

## 4. Discussion

In this prospective ED cohort of 41 patients undergoing IV thrombolysis for AIS, continuous bilateral frontal NIRS produced a physiological signal that paralleled the clinical course in real time. Three findings stand out. First, absolute rSO_2_ in the cohort as a whole was largely uninformative: pooled across all patients, it did not change in the unaffected hemisphere and rose only slightly in the affected hemisphere. Second, once patients were stratified by clinical trajectory, the affected hemisphere behaved in opposite ways—rising in those who improved and falling in those who deteriorated—and the interhemispheric ΔNIRS narrowed significantly, from 4 to 1, in the improvement group. Third, in the two patients who sustained an acute intra-treatment complication, bilateral desaturation was recorded before the clinical event; with only two cases, this observation is descriptive. One aspect must be stated plainly. Recanalisation and reperfusion were not assessed independently, so these findings show that NIRS tracked the clinical trajectory, not that it detected reperfusion itself.

The direction and magnitude of these changes are broadly consistent with the small body of work performed in the angiography suite. Ritzenthaler and colleagues, monitoring three patients during mechanical thrombectomy, found that the infarcted hemisphere started with a lower rSO_2_ and an interhemispheric difference exceeding 5%; when recanalisation succeeded, rSO_2_ on the infarcted side rose by approximately 10% and the interhemispheric gap closed by roughly 7%, whereas the failed procedure produced no change at all [10]. In a larger endovascular cohort, Hametner et al. observed sustained increases after recanalisation in only two of 43 patients, although a smaller burden of desaturation below baseline was associated with better reperfusion [11]. A post hoc analysis of the SIESTA trial found that successful recanalisation was followed by a small rise in affected-hemisphere oxygenation and a reduction in the interhemispheric difference [12]. In each of these studies, the optical changes could be tied to angiographically confirmed recanalisation. In the ED, no angiographic reference standard was available to us, so the parallel we describe is between NIRS and the clinical trajectory. It is compatible with pharmacological reperfusion but does not prove it. Ataş et al., in a Turkish ED cohort using an almost identical sampling schedule, likewise found a significant rise in affected-hemisphere rSO_2_ during IV thrombolysis in patients who improved clinically, and a significant correlation between changes in NIHSS and in affected-hemisphere rSO_2_ [17]; our results independently corroborate theirs, and add the mirror-image observation in patients who deteriorate.

Interhemispheric differences in NIRS signals have been described in acute stroke before and linked to the presence of large-vessel occlusion, to the success of recanalisation, and to the clinical course and to outcome [10,11,12,17,24,25,26]. Our study extends this work in four ways. Monitoring took place in the ED during IV thrombolysis before any endovascular procedure, sedation or anaesthesia, so the signal reflects the patient’s course under pharmacological treatment alone. Because all three clinical trajectories were examined, changes in opposite directions emerged in patients who improved and in those who deteriorated. Analysing each hemisphere separately revealed a between-group difference in the unaffected hemisphere, which we discuss below. Finally, we describe the behaviour of the signal around acute intra-treatment complications, which, to our knowledge, has not previously been reported during IV thrombolysis.

Not all groups have detected such changes. Hiramatsu et al. found NIRS unhelpful during thrombectomy for a distal middle cerebral artery occlusion even after complete recanalisation, while it performed well in large-vessel carotid occlusion [13], and attributed the discrepancy to lesion size and to the limited cortical volume interrogated by frontal optodes. This is not, in our view, a contradiction so much as a statement about what NIRS can and cannot see: a frontal optode samples a few centimetres of the anterior cortical mantle, so only ischaemia that alters oxygenation within that field will be registered. Over the forehead, the sampled field falls within the territory of the anterior cerebral artery and the anterior branches of the middle cerebral artery, including the anterior watershed between them [16]. The strokes most likely to be captured are large territorial middle cerebral artery infarcts and proximal internal carotid or middle cerebral artery occlusions, in which hypoperfusion reaches the frontal cortex and its watershed [11,13,25,27], together with infarcts in the anterior cerebral artery territory. By the same logic, distal branch occlusions confined to the temporal or parietal cortex, posterior cerebral artery territory infarcts, brainstem and cerebellar strokes, and small deep (lacunar) infarcts lie largely outside the optical field and are likely to produce false-negative recordings. It is therefore notable that our cohort was dominated by middle cerebral artery territory strokes (78%), which—through the anterior watershed and the lateral prefrontal cortex—do fall at least partly within the optical field.

We would draw particular attention to a finding that earlier work has largely overlooked: Our three trajectory groups differed significantly in the rSO_2_ of the unaffected hemisphere at every single time point. Patients who went on to improve had globally higher cerebral oxygenation from the outset, and those who deteriorated had globally lower values. One hypothesis follows: oxygenation of the unaffected hemisphere may carry prognostic information about the clinical course, reflecting, for example, collateral capacity and cerebrovascular reserve. The groups, however, were not balanced at baseline. Patients who deteriorated were older (mean 84.0 vs. 68.9 years in the improvement group), and four of the five were women (Table 1). In a large cohort, baseline rSO_2_ decreased with age and was lower in women and in patients with lower haemoglobin concentrations [19]. Absolute values are also influenced by skull thickness, by the cerebrospinal fluid layer [20], which widens with age-related atrophy, and by extracranial contamination [6,21]. In our deterioration group, whose patients were older and mostly women, the lower values may therefore reflect these patient-level factors rather than poorer cerebrovascular reserve. Our data cannot separate the two, and with only five patients in the group, adjusted analyses were not feasible. Whichever explanation holds, the methodological consequence is clear. Absolute rSO_2_ is confounded at the patient level and makes a poor candidate for a universal diagnostic threshold. ΔNIRS, by contrast, uses each patient as their own control and is less affected by these between-patient factors, although age-related differences in cerebrovascular reactivity could still influence how the signal changes over time. This is the central argument for reporting ΔNIRS rather than raw saturation, and it is in keeping with the interhemispheric-difference approach of earlier investigators [10,24]. Whether oxygenation of the unaffected hemisphere really predicts the clinical trajectory needs to be tested prospectively in a larger cohort, with adjustment for age, sex and haemoglobin concentration.

In the deterioration group, the median rSO_2_ of the unaffected hemisphere was lower than that of the affected hemisphere at every time point except 60 min (Table 3). This looks paradoxical, but it is not an artefact of comparing medians: within patients, the unaffected hemisphere had the lower value in three of the five patients at baseline and in two at 120 min (because ΔNIRS is an absolute difference, Table 4 shows the size of this gap but not its direction). Several factors may contribute. Two of the five patients also had bilateral desaturation during acute systemic events, which lowered the values on both sides. Physiology may contribute as well. Frontal rSO_2_ reflects the balance between oxygen delivery and consumption in the sampled cortex rather than the state of the infarct core, and reduced oxygen extraction by severely ischaemic or non-viable tissue can make rSO_2_ paradoxically higher over the infarcted hemisphere, as a previous series observed in most patients with complete middle cerebral artery infarction [25]. Chronic cerebrovascular disease in the nominally unaffected hemisphere, which is common in very elderly patients, could also have lowered the values on that side.

Continuous NIRS may also have a role in detecting acute systemic complications, although our evidence for this rests on two patients in our cohort. In the patient who had respiratory arrest, rSO_2_ fell bilaterally, reaching 19% and 31% at the moment of the event, and recovered after resuscitation; in the patient who seized, a fall preceded both the partial and the generalised event. The bilateral nature of these falls is potentially informative, because it may help to distinguish a global insult (hypoxaemia, apnoea, seizure-related metabolic demand, hypoperfusion) from the lateralised, unilateral changes that accompany reperfusion or extension of the infarct. This use of the modality is mechanistically plausible: NIRS is highly sensitive to acute changes in the balance between cerebral oxygen delivery and consumption, it picks up systemic desaturations at the stroke-unit bedside, particularly over the affected hemisphere [26], and multichannel NIRS has been used to assess dynamic cerebral autoregulation at the bedside in AIS [28]. We must be explicit, however, that two events in five patients constitute an observation, not evidence of predictive performance. No sensitivity, specificity or lead time can be estimated from these data, and any early-warning value of NIRS remains a hypothesis that requires prospective testing with prespecified alarm thresholds in a much larger cohort.

From an operational standpoint, the monitoring we studied is easy to adopt. NIRS devices are already present in many EDs, operating rooms and intensive care units; the optodes are adhesive, are applied in seconds and require no calibration, and did not interfere with the thrombolysis pathway in our study, where the median door-to-needle time was 59 min.

NIRS played no part in management in this study; monitoring was strictly observational. Should its value be confirmed, however, continuous NIRS could change the management of AIS in several ways. If affected-hemisphere rSO_2_ fails to rise during the infusion, or ΔNIRS widens, this could trigger earlier neurological reassessment and vascular imaging and support an earlier decision to proceed to, or transfer for, thrombectomy. Abrupt bilateral desaturation could prompt an immediate check of the airway, oxygenation, blood pressure and seizure activity. NIRS-derived indices of cerebral autoregulation could help to individualise blood pressure targets, an approach that has proved feasible in AIS [28,29]. Continuous monitoring might also prove useful during inter-hospital transfer after IV thrombolysis, when serial neurological examination is difficult. All of this is speculative. Each application would need to be shown to improve outcomes in interventional studies before NIRS could be recommended for routine use.

Future studies need to answer the questions that our design could not. ΔNIRS must first be validated against an independent reference for recanalisation and reperfusion, such as computed tomography or magnetic resonance perfusion imaging, angiography, or continuous transcranial Doppler monitoring, which can document recanalisation in real time during the infusion [30]. Combining frontal optical monitoring with CT perfusion imaging has already proved feasible during thrombectomy [27]. The discriminative performance of ΔNIRS then needs to be quantified, using receiver operating characteristic analysis with prespecified thresholds, in an adequately powered multicentre cohort with external validation. The optical signal should also be related to 24 h neurological status, symptomatic haemorrhage and 90-day functional outcome. On the technical side, multi-site optode arrays, short-separation channels to remove extracranial contamination, and time-domain or diffuse correlation spectroscopy may extend spatial coverage beyond the frontal cortex and improve the specificity of the signal [9,15,18,28].

### Limitations

Several limitations must be acknowledged. First, this was a single-centre observational study without a control group, and no a priori sample size calculation was performed. All consecutive patients were screened, and the seven who were not enrolled were missed for logistical reasons. Even so, the cohort was small; the deterioration group contained only five patients, so all analyses in that group are severely underpowered, and no change in ΔNIRS could be demonstrated. Multiple comparisons were performed across seven time points without formal adjustment, so isolated significant results may be chance findings. We did not attempt receiver operating characteristic analysis for the same reasons and because cut-off values selected in a data-driven way in small samples overestimate diagnostic accuracy [31]. Second, and most importantly, we had no angiographic or perfusion-imaging reference standard: recanalisation was never confirmed independently, so we can state that NIRS tracked the clinical trajectory, but not that it tracked successful recanalisation leading to reperfusion itself. The clinical trajectory groups were defined by NIHSS and GCS, and although the two scales were tightly correlated in our cohort, group allocation was not blinded to the investigators applying the monitor. Third, the trajectory groups were not balanced for age, and the proportion of women, although not significantly different, was highest in the deterioration group. Comorbidities and haemoglobin concentration, both of which may influence cerebral oximetry, were not part of the study dataset and could not be adjusted for. Fourth, measurements were confined to the hairless frontal region, which interrogates only the anterior cortical mantle; posterior circulation strokes and deep or lacunar infarcts are unlikely to be captured, and NIRS is prone to false-negative findings in small-vessel occlusions. Small (lacunar) infarcts that cause acute neurological symptoms are often missed on routine acute imaging, and diffusion-weighted magnetic resonance imaging remains the reference standard for confirming them [32]; our findings should not be extrapolated to such lesions. Fifth, frontal NIRS is subject to extracranial contamination from scalp and skull perfusion, which continuous-wave devices such as the INVOS cannot fully separate from the intracerebral signal [14,21]. Finally, monitoring stopped at 120 min and no functional outcome (modified Rankin Scale) was collected, so we cannot relate the optical signal to outcomes that matter to patients.

## 5. Conclusions

Continuous bilateral frontal NIRS during IV thrombolysis for AIS in the ED produced an objective, real-time physiological signal that paralleled the patient’s clinical trajectory: affected-hemisphere rSO_2_ rose, and the interhemispheric ΔNIRS narrowed in patients who improved, while affected-hemisphere rSO_2_ fell in those who deteriorated. Because absolute rSO_2_ differed between trajectory groups even in the structurally unaffected hemisphere, ΔNIRS—internally controlled and therefore less affected by this confounding—is the signal we would take forward. These findings show that NIRS tracked the clinical course during thrombolysis. They do not show that it detected recanalisation or reperfusion; neither was assessed. The bilateral desaturation seen in two patients with acute complications is a preliminary observation, not evidence of an early-warning role. These hypothesis-generating findings from a small, single-centre cohort justify a larger prospective study relating ΔNIRS to imaging-confirmed recanalisation and reperfusion and to functional outcome.

## Figures and Tables

**Table 1:** Baseline characteristics and process-of-care intervals, overall and by clinical trajectory.

Variable	All Patients (*n* = 41)	No Change (*n* = 21)	Improvement (*n* = 15)	Deterioration (*n* = 5)	*p*
Age, years, mean ± SD	73.6 ± 14.8	74.5 ± 14.5	68.9 ± 16.2	84.0 ± 3.0	0.002
Female sex, *n* (%)	26 (63.4)	12 (57.1)	10 (66.7)	4 (80.0)	0.732 *
Neurological severity at baseline, median (IQR)					
GCS	14 (11.5–15)	14 (11–15)	14 (12–15)	13 (12–15)	0.976
NIHSS	8 (6–14)	8 (6–14.5)	9 (6–14)	7 (6–12.5)	0.920
Process-of-care intervals, minutes, median (IQR)					
Door-to-imaging	22 (16–31.5)	22 (14.5–36.5)	22 (15–30)	32 (19.5–36.5)	0.437
Door-to-needle	59 (49.5–74)	59 (53.5–80)	59 (48–74)	60 (52.5–101)	0.550
Symptom onset-to-treatment	150 (125–190)	170 (135–205)	145 (100–180)	170 (127.5–185)	0.323
Baseline vital signs, mean ± SD					
Systolic BP, mmHg	156 ± 25	154 ± 25	153 ± 15	176 ± 41	0.509
Diastolic BP, mmHg	92 ± 16	89 ± 17	92 ± 10	101 ± 27	0.628
Heart rate, beats/min	88 ± 18	90 ± 17	83 ± 15	92 ± 28	0.438
Respiratory rate, breaths/min	18 ± 3	18 ± 3	19 ± 2	17 ± 4	0.638
Peripheral SpO_2_, %	94 ± 3	94 ± 2	95 ± 3	94 ± 3	0.809

BP, blood pressure; GCS, Glasgow Coma Scale; IQR, interquartile range; NIHSS, National Institutes of Health Stroke Scale; SD, standard deviation; Peripheral SpO_2_, peripheral oxygen saturation. * Comparison of the sex distribution between the three clinical trajectory groups.

**Table 2:** Vital signs of the whole cohort (*n* = 41) during thrombolysis, mean ± SD (minimum–maximum).

Time Point	Systolic BP (mmHg)	Diastolic BP (mmHg)	Heart Rate (/min)	Respiratory Rate (/min)	SpO_2_ (%)
Baseline	156 ± 25 (108–215)	92 ± 16 (62–130)	88 ± 18 (62–128)	18 ± 3 (12–24)	94 ± 3 (90–99)
0 min	156 ± 16 (128–179)	92 ± 15 (60–119)	89 ± 18 (56–127)	18 ± 2 (12–24)	95 ± 2 (91–99)
15 min	153 ± 20 (92–201)	90 ± 15 (60–116)	88 ± 17 (61–122)	18 ± 2 (12–24)	95 ± 2 (88–99)
30 min	152 ± 15 (124–187)	86 ± 15 (43–120)	87 ± 17 (58–129)	18 ± 2 (12–24)	95 ± 2 (90–99)
45 min	150 ± 22 (100–186)	88 ± 17 (60–140)	87 ± 18 (56–131)	18 ± 2 (12–24)	95 ± 2 (90–99)
60 min	147 ± 21 (112–189)	87 ± 13 (64–111)	88 ± 18 (58–120)	18 ± 4 (0–24)	95 ± 4 (74–99)
120 min	148 ± 22 (61–181)	86 ± 16 (32–114)	87 ± 17 (58–134)	18 ± 2 (12–24)	95 ± 3 (86–100)
*p* value	0.078	0.057	0.810	0.385	0.327

BP, blood pressure; SpO_2_, peripheral oxygen saturation.

**Table 3:** Regional cerebral oxygen saturation (rSO_2_, %) of the affected and unaffected hemispheres during thrombolysis, by clinical trajectory. Values are median (IQR).

Time Point	No Change (*n* = 21)		Improvement (*n* = 15)		Deterioration (*n* = 5)		*p* ^†^
	Affected	Unaffected ^†^	Affected	Unaffected ^†^	Affected	Unaffected ^†^	
Baseline	56 (49–62)	57 (47–65.5)	59 (57–68)	66 (61–70)	60 (53.5–70)	54 (45–69)	0.035
0 min	57 (47.5–62.5)	58 (49–64)	61 (59–66)	65 (61–70)	62 (55–67.5)	57 (47.5–67.5)	0.025
15 min	54 (46.5–62.5)	58 (49–64)	65 (58–73)	66 (61–72)	59 (48.5–66.5)	57 (47.5–66.5)	0.008
30 min	55 (47–62.5)	58 (53–65.5)	65 (59–72)	66 (61–69)	60 (45.5–67)	58 (46–66)	0.013
45 min	55 (48–62.5)	58 (52–63)	66 (59–72)	68 (62–70)	56 (44.5–64.5)	54 (47.5–66.5)	0.003
60 min	56 (49–65)	58 (53–63.5)	68 (60–74)	68 (60–70)	47 (32.5–61)	54 (36.5–64)	0.005
120 min	57 (50–64)	58 (53–65)	67 (61–70)	65 (61–74)	55 (51.5–64.5)	52 (48–64.5)	0.013
*p *^‡^ (over time)	0.090	0.638	0.001	0.939	0.015	0.956	

IQR, interquartile range; rSO_2_, regional cerebral oxygen saturation. ^†^ Between-group comparison (Kruskal–Wallis test) of the unaffected hemisphere at each time point. ^‡^ Within-group comparison across the seven time points (Friedman test).

**Table 4:** Absolute interhemispheric difference in cerebral oxygenation (ΔNIRS, |unaffected—affected|, %) at baseline and at 120 min by clinical trajectory. Values are median (IQR).

ΔNIRS	All Patients (*n* = 41)	No Change (*n* = 21)	Improvement (*n* = 15)	Deterioration (*n* = 5)
Baseline	4 (2–8)	3 (1–7.5)	4 (3–9)	4 (1–13.5)
120 min	2 (0.5–6.5)	3 (1–10)	1 (0–5)	5 (1–9.5)
*p* (baseline vs. 120 min)	0.114	0.886	0.034	0.285

IQR, interquartile range; ΔNIRS, absolute interhemispheric difference in regional cerebral oxygen saturation. Comparisons by Wilcoxon signed-rank test.

## Data Availability

The datasets generated and analysed during the current study are available from the corresponding author on reasonable request.

## Supplementary Materials

The following supporting information can be downloaded at https://www.mdpi.com/article/10.3390/diagnostics16213469/s1: Supplementary Table S1. Vital signs at each time point within each clinical trajectory group (mean ± SD, minimum–maximum). Supplementary Table S2. Glasgow Coma Scale and NIHSS at each time point, overall and by clinical trajectory group (median, IQR).

## References

[1] Powers W.J., Rabinstein A.A., Ackerson T., Adeoye O.M., Bambakidis N.C., Becker K., Biller J., Brown M., Demaerschalk B.M., Hoh B. (2019). Guidelines for the Early Management of Patients With Acute Ischemic Stroke: 2019 Update to the 2018 Guidelines for the Early Management of Acute Ischemic Stroke. Stroke.

[2] Emberson J., Lees K.R., Lyden P., Blackwell L., Albers G., Bluhmki E., Brott T., Cohen G., Davis S., Donnan G. (2014). Effect of treatment delay, age, and stroke severity on the effects of intravenous thrombolysis with alteplase for acute ischaemic stroke: A meta-analysis of individual patient data from randomised trials. Lancet.

[3] Brott T., Adams H.P., Olinger C.P., Marler J.R., Barsan W.G., Biller J., Spilker J., Holleran R., Eberle R., Hertzberg V. (1989). Measurements of acute cerebral infarction: A clinical examination scale. Stroke.

[4] Teasdale G., Jennett B. (1974). Assessment of coma and impaired consciousness. A practical scale. Lancet.

[5] Jöbsis F.F. (1977). Noninvasive, infrared monitoring of cerebral and myocardial oxygen sufficiency and circulatory parameters. Science.

[6] Ferrari M., Quaresima V. (2012). A brief review on the history of human functional near-infrared spectroscopy (fNIRS) development and fields of application. Neuroimage.

[7] Scheeren T.W.L., Schober P., Schwarte L.A. (2012). Monitoring tissue oxygenation by near infrared spectroscopy (NIRS): Background and current applications. J. Clin. Monit. Comput..

[8] Yang M., Yang Z., Yuan T., Feng W., Wang P. (2019). A systemic review of functional near-infrared spectroscopy for stroke: Current application and future directions. Front. Neurol..

[9] Moreau F., Yang R., Nambiar V., Demchuk A.M., Dunn J.F. (2016). Near-infrared measurements of brain oxygenation in stroke. Neurophotonics.

[10] Ritzenthaler T., Cho T.H., Luis D., Berthezene Y., Nighoghossian N. (2015). Usefulness of near-infrared spectroscopy in thrombectomy monitoring. J. Clin. Monit. Comput..

[11] Hametner C., Stanarcevic P., Stampfl S., Rohde S., Veltkamp R., Bösel J. (2015). Noninvasive cerebral oximetry during endovascular therapy for acute ischemic stroke: An observational study. J. Cereb. Blood Flow Metab..

[12] Batra D., Chen M., Meis J., Möhlenbruch M.A., Klose C., Ringleb P., Shah V., Bösel J., Schönenberger S. (2025). Feasibility of non-invasive neuromonitoring using BIS and NIRS during endovascular treatment of acute ischemic stroke. Neurol. Res. Pract..

[13] Hiramatsu R., Furuse M., Yagi R., Ohnishi H., Ikeda N., Nonoguchi N., Kawabata S., Miyachi S., Kuroiwa T. (2018). Limit of intraoperative near-infrared spectroscopy monitoring during endovascular thrombectomy in acute ischemic stroke. Interv. Neuroradiol..

[14] Collette S.L., Venema A.M., Eleveld N., Absalom A.R., Scheeren T.W., Verhoeve S., de Groot J.C., Elting J.W.J., Uyttenboogaart M., Bokkers R.P. (2022). Near-infrared spectroscopy monitoring during endovascular treatment for acute ischaemic stroke. Eur. Stroke J..

[15] Giacalone G., Zanoletti M., Re R., Germinario B., Contini D., Spinelli L., Torricelli A., Roveri L. (2019). Time-domain near-infrared spectroscopy in acute ischemic stroke patients. Neurophotonics.

[16] Annus Á., Nagy A., Vécsei L., Klivényi P. (2019). 24-hour near-infrared spectroscopy monitoring of acute ischaemic stroke patients undergoing thrombolysis or thrombectomy: A pilot study. J. Stroke Cerebrovasc. Dis..

[17] Ataş İ., Ersunan G., Bilir Ö., Yavaşi Ö., Altuntaş M., Karakullukçu S. (2024). The utility of NIRS in follow-up of patients with acute ischaemic stroke treated with IV thrombolysis and mechanical thrombectomy in the emergency department. J. Thromb. Thrombolysis.

[18] Delgado-Mederos R., Gregori-Pla C., Zirak P., Blanco I., Dinia L., Marín R., Durduran T., Martí-Fàbregas J. (2018). Transcranial diffuse optical assessment of the microvascular reperfusion after thrombolysis for acute ischemic stroke. Biomed. Opt. Express..

[19] Robu C.B., Koninckx A., Docquier M.-A., Grosu I., De Kerchove L., Mastrobuoni S., Momeni M. (2020). Advanced age and sex influence baseline regional cerebral oxygen saturation as measured by near-infrared spectroscopy: Subanalysis of a prospective study. J. Cardiothorac. Vasc. Anesth..

[20] Yoshitani K., Kawaguchi M., Miura N., Okuno T., Kanoda T., Ohnishi Y., Kuro M. (2007). Effects of hemoglobin concentration, skull thickness, and the area of the cerebrospinal fluid layer on near-infrared spectroscopy measurements. Anesthesiology.

[21] Davie S.N., Grocott H.P. (2012). Impact of extracranial contamination on regional cerebral oxygen saturation: A comparison of three cerebral oximetry technologies. Anesthesiology.

[22] Siegler J.E., Boehme A.K., Kumar A.D., Gillette M.A., Albright K.C., Martin-Schild S. (2013). What change in the National Institutes of Health Stroke Scale should define neurologic deterioration in acute ischemic stroke?. J. Stroke Cerebrovasc. Dis..

[23] Shi H.-X., Li C., Zhang Y.-Q., Li X., Liu A.-F., Liu Y.-E., Jiang W.-J., Lv J. (2023). Predictors of early neurological deterioration occurring within 24 h in acute ischemic stroke following reperfusion therapy: A systematic review and meta-analysis. J. Integr. Neurosci..

[24] Okune S., Marushima A., Koyama Y., Tanaka S., Araki K., Yoshimoto T., Hino T., Akimoto T., Hirata K., Hosoo H. (2025). Real-time monitoring of cerebrovascular insufficiency due to emergent large vessel occlusion using near-infrared spectroscopy based on the modified Beer-Lambert law: A retrospective study. World Neurosurg..

[25] Damian M.S., Schlosser R. (2007). Bilateral near infrared spectroscopy in space-occupying middle cerebral artery stroke. Neurocritical Care.

[26] Aries M.J., Coumou A.D., Elting J.W., van der Harst J.J., Kremer B.P., Vroomen P.C. (2012). Near infrared spectroscopy for the detection of desaturations in vulnerable ischemic brain tissue: A pilot study at the stroke unit bedside. Stroke.

[27] Favilla C.G., Forti R.M., Carter S., Kofke W.A., Kasner S.E., Baker W.B., Yodh A.G., Messé S.R., Cummings S., Kung D.K. (2024). Microvascular reperfusion during endovascular therapy: The balance of supply and demand. J. Neurointerv. Surg..

[28] Becker S., Klein F., König K., Mathys C., Liman T., Witt K. (2022). Assessment of dynamic cerebral autoregulation in near-infrared spectroscopy using short channels: A feasibility study in acute ischemic stroke patients. Front. Neurol..

[29] Petersen N.H., Silverman A., Strander S., Kodali S., Wang A., Sansing L.H., Schindler J.L., Falcone G.J., Gilmore E.J., Jasne A. (2020). Fixed compared with autoregulation-oriented blood pressure thresholds after mechanical thrombectomy for ischemic stroke. Stroke.

[30] Tsivgoulis G., Saqqur M., Sharma V.K., Brunser A., Eggers J., Mikulik R., Katsanos A.H., Sergentanis T.N., Perren F., Rubiera M. (2020). Timing of recanalization and functional recovery in acute ischemic stroke. J. Stroke..

[31] Leeflang M.M., Moons K.G., Reitsma J.B., Zwinderman A.H. (2008). Bias in sensitivity and specificity caused by data-driven selection of optimal cutoff values: Mechanisms, magnitude, and solutions. Clin. Chem..

[32] Thomas J.O., Visser M., Garcia-Esperon C., Spratt N.J., Cordato D., Cappelen-Smith C., Lin L., Parsons M.W. (2025). CT perfusion lesions are present in most MRI confirmed lacunar strokes. Neuroimage Clin..

