# Supplementary material for: Continuous Near-Infrared Spectroscopy as a Real-Time Monitor of Intravenous Thrombolysis in Acute Ischaemic Stroke: A Prospective Observational Emergency Department Study
Source: Diagnostics (Basel). 2026 Oct 23;16(21):3469. doi: 10.3390/diagnostics16213469 (PMC13761604; doi:10.3390/diagnostics16213469)
Supplement: Supplementary file 1 [file diagnostics-16-03469-s001.zip › diagnostics-4531255-supplementary.pdf]

Supplementary Material

Supplementary Table S1 Vital signs at each time point within each clinical trajectory group. Values are mean ± SD (minimum–maximum).

Time point	Systolic BP (mmHg)	Diastolic BP (mmHg)	Heart rate (/min)	Respiratory rate (/min)	SpO_2 (%)
**Clinical improvement (n = 15)**					
Baseline	153 ± 15 (121–182)	92 ± 10 (78–106)	83 ± 16 (63–120)	19 ± 2 (16–24)	95 ± 3 (90–99)
0 min	161 ± 12 (142–177)	94 ± 12 (60–106)	89 ± 16 (65–119)	19 ± 2 (16–24)	95 ± 2 (91–99)
15 min	152 ± 15 (126–174)	88 ± 15 (60–112)	88 ± 16 (70–122)	19 ± 2 (16–24)	95 ± 2 (92–99)
30 min	150 ± 11 (127–171)	86 ± 18 (43–116)	87 ± 15 (63–127)	19 ± 2 (16–24)	96 ± 2 (92–99)
45 min	149 ± 16 (120–172)	88 ± 11 (62–105)	88 ± 16 (61–120)	18 ± 2 (16–24)	95 ± 2 (93–98)
60 min	146 ± 19 (112–176)	85 ± 12 (64–110)	87 ± 15 (64–119)	19 ± 2 (16–24)	96 ± 2 (93–98)
120 min	150 ± 20 (108–179)	84 ± 12 (57–103)	88 ± 14 (60–116)	19 ± 2 (16–24)	95 ± 3 (90–99)
**No clinical change (n = 21)**					
Baseline	154 ± 25 (108–207)	89 ± 17 (62–130)	90 ± 17 (63–122)	18 ± 3 (12–22)	94 ± 2 (90–99)
0 min	151 ± 17 (128–179)	92 ± 16 (68–119)	90 ± 18 (56–127)	18 ± 2 (14–22)	95 ± 2 (91–98)
15 min	151 ± 16 (120–173)	92 ± 16 (61–116)	90 ± 16 (61–115)	18 ± 2 (14–22)	95 ± 2 (91–97)
30 min	151 ± 17 (124–187)	86 ± 14 (64–120)	88 ± 16 (58–113)	18 ± 2 (14–22)	95 ± 1 (92–99)
45 min	151 ± 25 (106–186)	91 ± 20 (62–140)	87 ± 17 (58–116)	18 ± 2 (14–22)	95 ± 2 (91–99)
60 min	147 ± 20 (112–181)	88 ± 13 (67–111)	89 ± 16 (59–115)	18 ± 2 (14–22)	95 ± 2 (92–99)
120 min	150 ± 16 (125–181)	89 ± 14 (67–109)	87 ± 15 (60–112)	19 ± 2 (14–22)	96 ± 2 (92–100)
**Clinical deterioration (n = 5)**					
Baseline	176 ± 41 (117–215)	101 ± 27 (62–130)	92 ± 28 (62–128)	17 ± 4 (12–22)	94 ± 3 (90–97)
0 min	160 ± 20 (129–179)	86 ± 17 (71–107)	84 ± 26 (64–113)	16 ± 4 (12–20)	94 ± 3 (92–98)
15 min	161 ± 41 (92–201)	84 ± 12 (73–103)	80 ± 25 (63–121)	16 ± 4 (12–20)	93 ± 3 (88–97)
30 min	165 ± 16 (147–180)	87 ± 14 (67–99)	81 ± 28 (58–129)	16 ± 4 (12–20)	94 ± 3 (90–97)
45 min	150 ± 32 (100–184)	77 ± 16 (60–100)	86 ± 32 (56–131)	17 ± 3 (12–20)	94 ± 3 (90–97)
60 min	153 ± 34 (118–189)	85 ± 16 (68–108)	83 ± 30 (58–120)	13 ± 8 (0–20)	90 ± 9 (74–97)
120 min	129 ± 42 (61–173)	74 ± 31 (32–114)	85 ± 30 (58–134)	16 ± 3 (12–20)	94 ± 4 (86–97)

BP, blood pressure; SD, standard deviation; SpO_2, peripheral oxygen saturation.

Supplementary Table S2 Glasgow Coma Scale and NIHSS at each time point. Values are median (IQR).

Time point	All patients (n = 41)		No change (n = 21)		Improvement (n = 15)		Deterioration (n = 5)	
	GCS	NIHSS	GCS	NIHSS	GCS	NIHSS	GCS	NIHSS
Baseline	14 (11.5–15)	8 (6–14)	14 (11–15)	8 (6–14.5)	14 (12–15)	9 (6–14)	13 (12–15)	7 (6–12.5)
0 min	14 (11.5–15)	8 (6–14)	14 (11–15)	8 (6–14.5)	14 (12–15)	9 (6–14)	13 (12–15)	7 (6–13.5)
15 min	14 (11.5–15)	8 (6–14)	14 (11–15)	8 (6–14.5)	14 (13–15)	8 (6–14)	13 (11.5–15)	8 (7–13.5)
30 min	14 (11.5–15)	8 (6–14)	14 (11–15)	8 (6–14.5)	14 (13–15)	8 (6–14)	13 (11.5–15)	8 (7–14.5)
45 min	14 (11–15)	8 (5.5–14)	14 (11–15)	8 (5.5–14.5)	14 (13–15)	7 (5–14)	13 (10.5–15)	13 (7.5–15)
60 min	14 (11–15)	8 (5–13.5)	14 (11–15)	8 (5.5–14)	15 (14–15)	5 (4–12)	11 (6.5–14)	14 (10.5–16.5)
120 min	15 (11–15)	8 (5–13.5)	14 (11–15)	8 (5.5–14)	15 (14–15)	5 (3–12)	11 (7–14)	14 (10.5–15.5)

GCS, Glasgow Coma Scale; IQR, interquartile range; NIHSS, National Institutes of Health Stroke Scale. GCS and NIHSS were strongly inversely correlated at every time point in the whole cohort (Spearman $\rho = -0.740$ at baseline, −0.742 at 0 min, −0.800 at 15 min, −0.809 at 30 min, −0.748 at 45 min, −0.844 at 60 min, −0.820 at 120 min; all $p < 0.001$).
